# Subthalamic deep brain stimulation for primary dystonia: defining an optimal location using the medial subthalamic nucleus border as anatomical reference

**DOI:** 10.3389/fnagi.2023.1187167

**Published:** 2023-07-21

**Authors:** Mingming Zhao, Hui Chen, Xin Yan, Jianguang Li, Chao Lu, Bin Cui, Wenjun Huo, Shouming Cao, Hui Guo, Shuang Liu, Chunjuan Yang, Ying Liu, Feng Yin

**Affiliations:** ^1^Department of Neurosurgery, Aerospace Center Hospital, Beijing, China; ^2^Department of Radiology, Aerospace Center Hospital, Beijing, China

**Keywords:** dystonia, deep brain stimulation, subthalamic nucleus, movement disorders, neurological function

## Abstract

**Introduction:**

Although the subthalamic nucleus (STN) has proven to be a safe and effective target for deep brain stimulation (DBS) in the treatment of primary dystonia, the rates of individual improvement vary considerably. On the premise of selecting appropriate patients, the location of the stimulation contacts in the dorsolateral sensorimotor area of the STN may be an important factor affecting therapeutic effects, but the optimal location remains unclear. This study aimed to define an optimal location using the medial subthalamic nucleus border as an anatomical reference and to explore the influence of the location of active contacts on outcomes and programming strategies in a series of patients with primary dystonia.

**Methods:**

Data from 18 patients who underwent bilateral STN-DBS were retrospectively acquired and analyzed. Patients were assessed preoperatively and postoperatively (1 month, 3 months, 6 months, 1 year, 2 years, and last follow-up after neurostimulator initiation) using the Toronto Western Spasmodic Torticollis Rating Scale (for cervical dystonia) and the Burke–Fahn–Marsden Dystonia Rating Scale (for other types). Optimal parameters and active contact locations were determined during clinical follow-up. The position of the active contacts relative to the medial STN border was determined using postoperative stereotactic MRI.

**Results:**

The clinical improvement showed a significant negative correlation with the y-axis position (anterior–posterior; A+, P−). The more posterior the electrode contacts were positioned in the dorsolateral sensorimotor area of the STN, the better the therapeutic effects. Cluster analysis of the improvement rates delineated optimal and sub-optimal groups. The optimal contact coordinates from the optimal group were 2.56 mm lateral, 0.15 mm anterior, and 1.34 mm superior relative to the medial STN border.

**Conclusion:**

STN-DBS was effective for primary dystonia, but outcomes were dependent on the active contact location. Bilateral stimulation contacts located behind or adjacent to Bejjani’s line were most likely to produce ideal therapeutic effects. These findings may help guide STN-DBS preoperative planning, stimulation programming, and prognosis for optimal therapeutic efficacy in primary dystonia.

## 1. Introduction

Dystonia is a movement disorder characterized by sustained or intermittent muscle contractions that result in abnormal movements and/or posture (Balint and Bhatia, 2014). Treatment is challenging because of dystonia’s highly complex etiology and pathogenesis (Batla et al., 2012; Balint et al., 2018; Albanese et al., 2019). Deep brain stimulation (DBS) has been widely used in the treatment of various types of drug-resistant dystonia, and the globus pallidus internus (GPi) is the most commonly used stimulation target (Kupsch et al., 2006; Volkmann et al., 2014; Meoni et al., 2017; Sobstyl et al., 2017; Tsuboi et al., 2020). Although its safety and efficacy have been proven, stimulation-induced side effects are frequent and insurmountable (Balint et al., 2018; Kosutzka et al., 2020). The high energy consumption of DBS is another drawback (Lin et al., 2019).

Increasing evidence has shown that the subthalamic nucleus (STN) is an effective target for the treatment of primary dystonia (Yao et al., 2019; Wang and Yu, 2021; Li et al., 2022; Yin et al., 2022). However, the benefits of stimulation and the required stimulation complexity vary greatly between patients, highlighting the necessity of exploring outcome and treatment predictors. The main factors affecting the success of STN-DBS include patient selection, the correct positioning of the electrodes in the target, and the optimization of stimulation programming. Choosing the optimal DBS site is a prerequisite for obtaining good therapeutic effects.

The midcommissural point (MCP) is a common anatomical reference point used in stereotactic neurosurgery. However, using this for STN-DBS localization is not ideal, as the exact location of the MCP varies among individuals (Bot et al., 2018). The red nucleus (RN) is a useful internal reference for targeting the anteroposterior coordinates of the STN (Chang et al., 2008). Bejjani’s line (Bejjani et al., 2000) and the medial STN border, based on the anatomic relationship between the RN and STN, have also been used with good results in STN-DBS treatment of Parkinson’s disease (PD), and a theoretical stimulation “hotspot” has been defined (Bot et al., 2018). However, the usefulness of this location in dystonia remains unclear. Therefore, we used the medial border of the STN as a reference to determine the optimal electrode location and to evaluate the relationship between active contact locations, clinical outcomes, and programming in the STN-DBS treatment of primary dystonia.

## 2. Materials and methods

### 2.1. Patients

We retrospectively analyzed data collected from 18 patients with primary dystonia who received STN-DBS in the Department of Neurosurgery at the Aerospace Center Hospital from September 2014 to January 2020. The inclusion criteria were as follows: a diagnosis of idiopathic isolated dystonia; severe dysfunction that did not respond to oral medication, botulinum toxin, or selective peripheral denervation; no other secondary cause, including the use of antipsychotic medications, was present before the onset of dystonia; normal neurological examination and brain MRI except for dystonia; and the patient was willing to receive regular counseling visits and a long-term follow up. The exclusion criteria were medical contraindications to surgery; MRI evidence of another neurological disorder, extensive brain atrophy, or anatomic abnormalities in the basal ganglia region; and severe cognitive impairment, depression, or severe mental illness. This study received ethical approval from the Aerospace Center Hospital (approval number: 20190301-YN-03), and all protocols were implemented in accordance with the Declaration of Helsinki. All patients provided written informed consent.

### 2.2. Surgical procedures and stimulation programming

The dorsolateral regions of the bilateral STN were selected as the targets for electrode implantation in all enrolled patients. DBS surgery was performed by the same two experienced neurosurgeons following a previously published procedure (Yin et al., 2022). All patients underwent post-operative brain CT to rule out hemorrhage. Programming was initiated 3 weeks after DBS surgery. The lead locations were confirmed by fusing post-operative high-resolution CT images with pre-operative MRI before programming. The programming method has also been described previously (Yin et al., 2022).

### 2.3. Clinical evaluation

Symptoms of dystonia were assessed by an independent neurologist specializing in movement disorders, who was neither aware of the stimulation status nor responsible for programming, before (baseline) and after surgery (1 month, 3 months, 6 months, 1 year, 2 years, and at the last follow-up after neurostimulator initiation). The Toronto Western Spasmodic Torticollis Rating Scale was used to assess cervical dystonia (CD), and the Burke–Fahn–Marsden Dystonia Rating Scale (BFMDRS) was used to assess generalized dystonia, cranial dystonia, and myoclonus–dystonia. The results were normalized by calculating the percentage changes of both rating scale scores.

### 2.4. Electrode contact placement relative to medial STN border

The methods of Bot et al. (2018) were followed for electrode contact positioning relative to the medial STN border. In brief, 1.5-T T2-weighted MRI was performed, and measurements were performed using SurgiPlan. The medial STN border was identified in the axial plane containing the maximum diameter of the RN, which was determined using both axial- and coronal-orientated images. A line was drawn perpendicular to the anterior commissure–posterior commissure line coinciding with the anterior border of the RN, which is Bejjani’s line. The point of intersection with the medial boundary of the STN was determined, defined as the medial STN border, and the stereotactic coordinates with respect to the MCP were recorded. Post-operative CT images were coregistered with stereotactic T1-weighted MRI images, and the stereotactic x- (lateral), y- (anterior–posterior), and z- (dorsal–ventral) coordinates of the contact point of active stimulation relative to the medial STN border were determined. This was done separately for the left and right hemispheres. The x-coordinates of both bilateral contacts were defined as the location of the positive contact, and the y- and z-coordinates were used to define the anterior and dorsal directions of Bejjani’s line as positive and the reverse as negative.

### 2.5. Statistical analysis

Statistical analysis was performed using SPSS (v19.0; IBM Corp., Armonk, NY, USA). The Shapiro–Wilk test was used to analyze the distribution of the grouped data. Cluster analysis (K-means clustering) was used to identify subgroups using improvement rates. The Mann–Whitney U test was used to compare differences between clusters, between dystonia subtypes, and between coordinate values. Correlations were performed using Spearman’s correlation analysis. Two-tailed *p*-values < 0.05 were considered statistically significant. The results are presented as mean ± SD.

## 3. Results

### 3.1. Participants

Table 1 summarizes the clinical characteristics, percentages of improvement at different follow-up times, and stimulation parameters at the last follow-up of the 18 included patients (9 male, 9 female). Nine patients had CD, five had generalized dystonia, three had cranial dystonia (one with cervical symptoms and two without), and one had myoclonus–dystonia. The mean age of onset was 39.3 ± 15.1 (range, 7–62) years. The duration of disease was 4.4 ± 2.2 (range, 1–9) years. The average age at surgery was 43.7 ± 14.9 (range, 14–69) years. The mean follow-up time was 5.5 ± 1.8 (range, 2–8) years. Two patients received routine battery replacements.

**TABLE 1:** Summary of patient characteristics, percent improvement at different follow-up times, and stimulation parameters at last follow-up.

Sex	
Male	9
Female	9
Age at onset (year)	39.3 ± 15.1
Childhood	1
Adolescence	1
Early adulthood	7
Late adulthood	9
Disease duration (year)	4.4 ± 2.2
Disease subtype	
Generalized	5
Cervical	9
Cranial	3
Myoclonus	1
Age at surgery (year)	43.7 ± 14.9
Duration of follow-up (year)	5.5 ± 1.8
Percentage of improvement	
1 month	23.8 ± 10.9
3 months	52.3 ± 17.2
6 months	69.4 ± 25.6
1 year	83.0 ± 22.8
2 years	85.7 ± 23.6
Last follow-up	90.6 ± 13.0
DBS parameters	
Amplitude (V)	2.4 ± 0.5
Pulse width (μs)	60.6 ± 2.3
Frequency (Hz)	134.0 ± 6.7

Data on age at onset, disease duration, age at surgery, duration of follow-up, percentage of improvement, and DBS parameters expressed as mean ± SD, and other data expressed as numbers; DBS, deep brain stimulation.

### 3.2. Clinical outcomes

**FIGURE 1:**
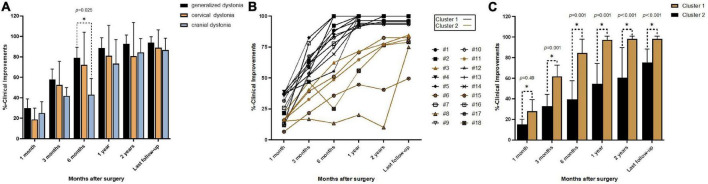
Improvement rates at different follow-up times: **(A)** mean percent improvement at different follow-up times after surgery in three types of dystonia; **(B)** line graphs showing individual percent improvement at different follow-up times after surgery; **(C)** mean percent improvement at different follow-up times after surgery in Clusters 1 and 2; *p < 0.05.

A total of 36 DBS electrodes were placed in 18 patients, and all used the monopolar stimulation mode. For the entire cohort, the mean improvement was 23.8% at 1 month, 52.3% at 3 months, 69.4% at 6 months, 83.0% at 1 year, 85.7% at 2 years, and 90.6% at the last follow-up. The mean improvement rates of five patients with generalized dystonia at 1, 3, and 6 months and 1 year, 2 years, and the last follow-up were 29.9, 57.9, 79.2, 88.8, 92.6, and 94.0%, respectively. Correspondingly, in the nine patients with CD, these were 18.7, 52.6, 72.1, 81.0, 80.8, and 89.0%, respectively. In the three patients with cranial dystonia, these were 25.1, 41.9, 43.0, 73.5, 84.3, and 86.8%, respectively. There was no significant difference among the three types of dystonia during follow-up except for a slight difference between generalized and cranial dystonia at 6 months (Figure 1A). The patient with myoclonus–dystonia showed improvement rates at 1 month, 3 months, 6 months, 1 year, 2 years, and the last follow-up of 35.7, 52.4, 74.7, 100.0, 100.0, and 100.0%, respectively.

At the last follow-up visit, all the patients received a monopolar stimulus mode. The mean pulse width was 60.6 ± 2.3 μs, the mean frequency was 134.0 ± 6.7 Hz, and 17 patients were using constant-voltage stimulation (mean amplitude, 2.4 ± 0.5 V) and 1 patient was using constant-current stimulation (bilateral, 2.4 mA).

Cluster analysis of the improvement rates identified two different groups (Figures 1B, C). Cluster 1 included 12 patients (6 CD, 4 generalized dystonia, 1 cranial dystonia, and 1 myoclonus–dystonia), and the mean improvement rates at 1 month, 3 months, 6 months, 1 year, 2 years, and the last follow-up were 28.1, 61.9, 84.4, 97.2, 98.3, and 98.3%, respectively. Cluster 2 included six patients (3 CD, 1 generalized dystonia, and 2 cranial dystonia), and the improvement rates were 15.3, 32.9, 39.4, 54.6, 60.6, and 75.3%, respectively. The mean improvement was statistically different between these two groups at each follow-up time. Cluster 1 represents the optimal response group, and Cluster 2 represents the suboptimal response group. The two groups had no significant differences in sex (*p* = 0.331), age at disease onset (*p* = 0.174), duration of disease (*p* = 0.479), age at surgery (*p* = 0.189), duration of stimulation (*p* = 0.743), and stimulation parameters (left amplitude, *p* = 0.850; right amplitude, *p* = 0.395; left pulse width, *p* = 1.000; right pulse width, *p* = 0.606; frequency, *p* = 0.538).

### 3.3. Location of active electrode contacts

The mean stereotactic distances of the left and right active electrode contacts of Cluster 1 and Cluster 2 relative to the medial STN border are shown in Table 2. The active contacts in Cluster 2 were more anterior than those in Cluster 1 on both the left and right sides, but there was no significant difference in the x- or z- coordinates. In both clusters, there was no significant difference between the right and left sides for the x-, y-, or z- coordinates. For Cluster 1, the average x-, y-, and z-coordinates were 2.56 mm, 0.15 mm, and 1.34 mm, respectively. The optimal contact coordinates were obtained according to these. For Cluster 2, the average x-, y-, and z-coordinates were 2.66 mm, 1.48 mm, and 1.08 mm, respectively. Cluster 1 and Cluster 2 were statistically different in their average y-coordinates but not in their average x- or z-coordinates (Table 2).

**TABLE 2:** Mean stereotactic coordinates of left and right active electrode contacts in Cluster 1 and Cluster 2 relative to the medial STN border and average coordinates on both sides.

	*n*	Left contact location			Right contact location			Average coordinates of bilateral contact location		
		*x*	*y*	*z*	*x*	*y*	*z*	*x*	*y*	*Z*
Cluster 1	12	2.65 ± 0.33	−0.05 ± 0.65	1.28 ± 1.33	2.47 ± 0.3	0.36 ± 0.59	1.87 ± 1.04	2.56 ± 0.22	0.15 ± 0.60	1.34 ± 1.24
Cluster 2	6	2.55 ± 0.44	1.02 ± 0.67	0.3 ± 1.61	2.77 ± 0.3	1.93 ± 0.6	1.4 ± 1.26	2.66 ± 0.36	1.48 ± 0.57	1.08 ± 0.86
*P*-values		*p* = 0.741	***p* = 0.017**	*p* = 0.280	*p* = 0.066	***p* = 0.001**	*p* = 0.606	*p* = 0.280	***p* = 0.001**	*p* = 0.398

Significant differences between clusters indicated in bold.

**FIGURE 2:**
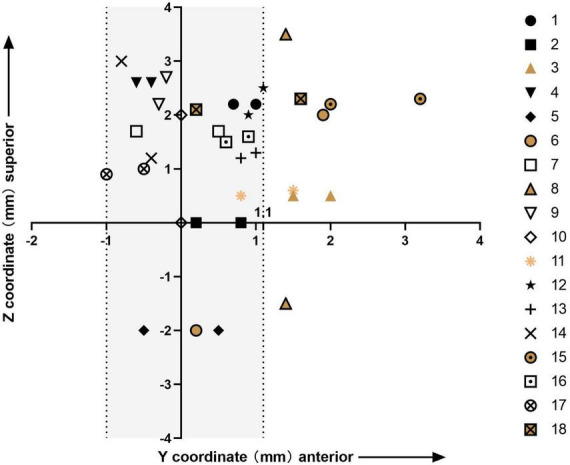
Plot of the y- and z-coordinates of the bilateral stimulation contacts for all 18 patients; the origin of the plot is the medial STN border; Clusters 1 and 2 are represented in different colors; the shaded area includes all bilateral active contacts of patients in Cluster 1 and unilateral active contacts of patients in Cluster 2.

The bilateral active contacts in all the patients are shown in Figure 2. In Cluster 2, both bilateral active contacts were more anterior in three patients, and the active contacts on one side were more anterior than the other side in three patients. The former group showed less improvement at the last follow-up than the latter group (69.7 vs. 80.9%), and the small number of cases limited statistical analysis.

### 3.4. Correlation between active contacts locations and outcomes

**FIGURE 3:**
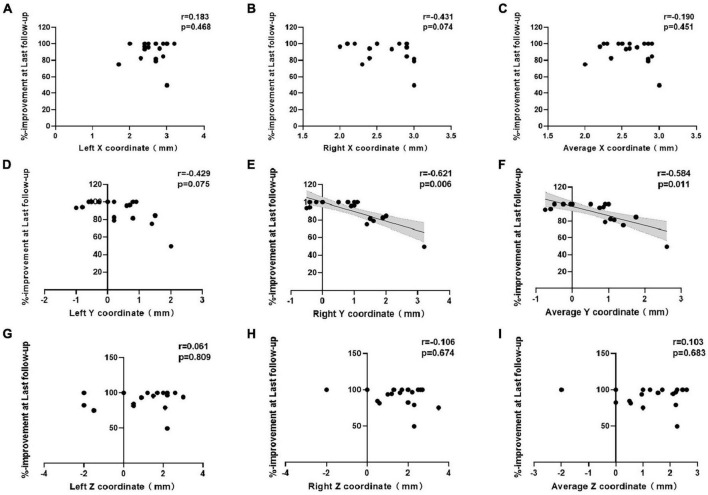
Correlations between percent improvement at last follow-up and the **(A–C)** x-, **(D–F)** y-, and **(G–I)** z-coordinates of the active contacts in all patients.

Correlations between the improvement rate at the last follow-up and the x-, y-, and z-coordinates relative to the medial STN border are shown in Figure 3. For the entire cohort, there was a significant inverse correlation between the right y-coordinate and the improvement rate at the last follow-up (*p* = 0.006). The improvement rate at the last follow-up showed no correlation with the left y-, bilateral x-, or bilateral z- coordinates. The average y-coordinate of the bilateral contacts was negatively correlated with the improvement rate at the last follow-up (*p* = 0.011), while the average x- and z- coordinates were not significantly correlated with the improvement rate at the last follow-up.

### 3.5. Long-term motor outcome predictors and associated factors

None of the tested factors were found to be an independent predictor of long-term movement improvement (sex, *p* = 0.281; age at disease onset, *p* = 0.061; duration of disease, *p* = 0.793; age at surgery, *p* = 0.057; duration of stimulation, *p* = 0.163). However, the improvement rates at 1 month (*p* = 0.022) and 3 months (*p* = 0.001) were positively correlated with the improvement rate at the last follow-up.

### 3.6. Adverse events

No surgery-related complications (e.g., intracranial and extracranial hematoma or epileptic seizures) or hardware-related infections were found during the entire follow-up. Six patients experienced uncomfortable sensations due to the extension wire, but none required additional surgery. One patient sometimes experienced mild pain at the site of the neurostimulator, but it had no practical effect on activities of daily living. Stimulus-related adverse events included mild balance disorder (one patient), manic symptoms (one patient), mild hand weakness (two patients), and movement disorders (10 patients), all of which were alleviated through programming alterations.

## 4. Discussion

This study has once again confirmed the long-term safety and sustained effectiveness of STN-DBS for the treatment of different subtypes of dystonia (up to 8 years). We determined the optimal contact coordinates, which were relative to the medial STN border, for STN-DBS in the treatment of dystonia and found that the improvement of symptoms was closely related to the y-axis position of the electrode contact. The more posterior the electrode contacts were in the dorsolateral sensorimotor area of the STN, the better the therapeutic effects. This study demonstrated significant effects of STN-DBS in the treatment of myoclonus–dystonia.

Treatment with STN-DBS is more likely to induce stimulus-related dyskinesia than GPi-DBS, and this most often occurs in the early stages after the stimulator is first activated (Zheng et al., 2010; Lin et al., 2019; Liu et al., 2019). We reduced the voltage within 1 month after neurostimulator initiation to prevent discomfort and lower the risk of adverse events. If the treatment threshold was not reached, the improvement rate at the 1-month follow-up was lower. Then, by gradually increasing the stimulation voltage, the dyskinesia was overcome, and the treatment effect gradually became significant. The same strategy was used by our group for the treatment of all types of dystonia.

Cervical dystonia is the most common form of focal dystonia, and there are more reports on STN-DBS treatment of CD than other subtypes (Pahapill and O’Connell, 2010; Ostrem et al., 2011, 2017; Wagle Shukla et al., 2018; Gupta, 2020). In the present study, long-term improvement was higher than in a previous study (Ostrem et al., 2017). Our current study included five patients with generalized dystonia, who showed no significant difference in their mean motor symptom improvement rate compared with patients with CD during follow-up; this was in line with a previous study (Deng et al., 2018). A recent study showed that STN-DBS provided relatively steady improvement in the severity of generalized isolated dystonia, with increases of 66.8 and 72.6% at 1-year and last long-term follow-up, respectively (Li et al., 2022). In the three patients in the present study with cranial dystonia, the mean improvement rate was not significantly different from that in the patients with CD and generalized dystonia. A recent study showed that 32 patients with Meige syndrome had a mean improvement of 79.0% at the last follow-up (mean, 16.3 months; Wang et al., 2021), which is similar to that reported here. Another study showed that 14 patients with Meige syndrome had a mean improvement of 70.9% at the last follow-up (mean, 14.8 months; Yao et al., 2019).

Myoclonus–dystonia is a relatively rare movement disorder typically characterized by childhood-onset myoclonic jerks in the upper limbs and various extents of dystonia (Roze et al., 2018). Most studies have selected GPi as the stimulation target, and few have chosen the ventral intermediate nucleus of the thalamus. Both targets have been effective, but GPi stimulation may be preferred due to fewer stimulation-induced events (Wang and Yu, 2021). In the present study, we included one patient with myoclonus–dystonia who achieved complete improvement 1 year after STN-DBS. Similar reports have not been found.

Studies of STN-DBS for the treatment of PD, when the MCP was selected as the reference, have shown no correlation between the DBS location and motor improvement (McClelland et al., 2005, 2009; Kasasbeh et al., 2013; Weise et al., 2013; Nestor et al., 2014). Bot et al. (2018) proposed the medial STN border, which was defined as the intersection of Bejjani’s line with the medial border of the STN (Bejjani et al., 2000), as a new, individualized reference point that is well delineated on standard MRI (Bot et al., 2018). They found that the medial STN border was superior compared to the MCP as an anatomical reference for correlation between the DBS location and motor improvement and defined a theoretic stimulation “hotspot.” A study with a larger patient cohort study refined the “hotspot” within the STN at 2.6 mm lateral, 0.7 mm anterior, and 1.9 mm superior to the medial STN border using T2-weighted imaging (Bolier et al., 2021). Inspired by this, in the present study, we found that the “hotspot” for STN-DBS in the treatment of primary dystonia was at 2.56 mm lateral, 0.15 mm anterior, and 1.34 mm superior to the medium STN border using T2-weighted imaging. The “hotspots” for the treatment of dystonia and PD are, thus, similar. The subsequent findings of an exclusive correlation between the y-coordinate and clinical outcome suggested that the y-axis placement was an important predictor of electrode contact efficacy.

The explanation for these findings may relate to the anatomy of the STN. The STN is composed of a dorsolateral motor area, a central associative region, and a ventromedial limbic component (Haynes and Haber, 2013). The more forward the electrode contact deviates from Bejjani’s line, the closer it is to the associative region, and it can thus can easily cause stimulation side effects. The dorsolateral motor area neurons are then less stimulated, resulting in a poor therapeutic effect.

In the present study, Cluster 1 represented the optimal response group, and Cluster 2 represented the suboptimal response group. During the entire follow-up period, the improvement rate of Cluster 1 was significantly better than that of Cluster 2. Interestingly, the improvement of three patients from Cluster 2, with active contacts on one side more anterior to those on the other, was superior to the three patients for whom both bilateral active contacts were more anterior, although the number of cases limited statistical analysis. This may mean that the optimal therapeutic effect requires bilateral stimulation contacts to be in an ideal location. Even if one of the stimulation contacts deviates from this position, the long-term outcome may be affected. The present study also confirmed that axial symptoms of dystonia are regulated bilaterally in the brain. The electrode deviation may have been related to brain drift caused by the loss of cerebrospinal fluid (CSF) during implantation, and the second electrode is especially more vulnerable to this. Direct puncture of the dura over the planned cortical entry point during surgery can significantly reduce the loss of CSF compared to standard incision of the dura (Piacentino et al., 2021).

The “up–top–down” rule is the programming strategy we apply, as described in our previous article, which minimizes stimulus-related side effects and reduces the energy consumption of the stimulator while maintaining efficacy (Yin et al., 2022). No significant difference was detected between Cluster 1 and Cluster 2 in the incidence of stimulation-induced dyskinesia in the present study, but Cluster 1 was less severe and easier to adapt than Cluster 2. This may be related to the better contact positions in Cluster 1.

In the present cohort, the optimal selection of active contacts was determined within 3 months for Cluster 1, and with the increase of the stimulation voltage, motor symptoms continued to improve until becoming stable. However, for Cluster 2, the mean improvement rate at 3 months was less than that in Cluster 1 (32.9 vs. 62.9%). In order to obtain better therapeutic effects, it is necessary to alter the active contacts and/or stimulation mode after 3 months of stimulation. In the present study, one patient’s paroxysmal dystonia disappeared immediately after switching from constant-voltage to constant-current stimulation. The improvement rate at 3 months could predict the effects of long-term stimulation, and using the 3-month rate for prediction was superior to the 1-month rate. Thus, when the improvement rate is not ideal after 3 months, an adjustment of the stimulation strategy should be considered.

One patient in the present study became hypomanic after the stimulator was turned on, indicating that the stimulation contact was located in the ventral limbic region (Mallet et al., 2007; Prange et al., 2022). When the dorsal contacts were selected as the active contacts, the hypomania disappeared, but the stimulation effect was poor because the stimulation contacts deviated from Bejjani’s line on both sides.

This study has some limitations. First, it was not randomized or fully blinded, which may have introduced bias. Future studies are warranted that group patients randomly, by differentiation using the stimulation electrode y-coordinates, or using blind clinical assessment, which will help confirm our findings. Second, the analysis of the predictors may not be robust enough due to a lack of sufficient case numbers. Therefore, more cases and more rigorously designed studies are needed for further confirmation. Genetic testing data were not available for most of the patients in the present cohort. A growing number of studies have found that genetic signatures are some of the most promising predictors (Jinnah et al., 2017; Tisch and Kumar, 2020).

## 5. Conclusion

Subthalamic nucleus-deep brain stimulation can provide significant, sustained, and stable effects in the treatment of patients with various subtypes of primary dystonia. In addition, stimulation at posterior contacts in the STN on the y-axis was found to be more advantageous than at anterior contacts for improving dystonia. Special attention should be paid to electrode positioning along the anterior–posterior axis to ensure that the electrodes are positioned as close to Bejjani’s line as possible.

## Data availability statement

The raw data supporting the conclusions of this article will be made available by the authors, without undue reservation.

## Ethics statement

This study was approved by the Ethics Committee of the Aerospace Center Hospital (Approval Number: 20190301-YN-03). Written informed consent to participate in this study was provided by the participants’ legal guardian/next of kin.

## Author contributions

MZ, FY, HC, and CL conceptualized the study. MZ, JL, CY, and YL conducted the study, including data collection and data analysis. XY and SC contributed to the assessment of pre- and post-operative symptoms. BC, HG, and WH contributed to the acquisition and processing of image data. MZ and SL drafted the manuscript, which was critically reviewed by all the other authors. All the authors approved the final version of the manuscript.
